# Integration of interictal EEG source localization in presurgical epilepsy evaluation – A single‐center prospective study

**DOI:** 10.1002/epi4.12754

**Published:** 2023-05-19

**Authors:** Gadi Miron, Thomas Baag, Kara Götz, Martin Holtkamp, Bernd J. Vorderwülbecke

**Affiliations:** ^1^ Epilepsy‐Center Berlin‐Brandenburg Institute for Diagnostics of Epilepsy Berlin Germany; ^2^ Department of Neurology, Epilepsy‐Center Berlin‐Brandenburg Charité – Universitätsmedizin Berlin Berlin Germany

**Keywords:** drug‐resistant focal epilepsy, electric source imaging, epilepsy surgery

## Abstract

**Objective:**

To investigate cost in working hours for initial integration of interictal EEG source localization (ESL) into clinical practice of a tertiary epilepsy center, and to examine concordance of results obtained with three different ESL pipelines.

**Methods:**

This prospective study covered the first year of using ESL in the Epilepsy‐Center Berlin‐Brandenburg. Patients aged ≥14 years with drug‐resistant focal epilepsy referred for noninvasive presurgical evaluation were included. Interictal ESL was based on low‐density EEG and individual head models. Source maxima were obtained from two freely available software packages and one commercial provider. One physician and computer scientist documented their working hours for setting up and processing ESL. Additionally, a survey was conducted among epilepsy centers in Germany to assess the current role of ESL in presurgical evaluation.

**Results:**

Of 40 patients included, 22 (55%) had enough interictal spikes for ESL. The physician's working times decreased from median 4.7 hours [interquartile range 3.9‐6.4] in the first third of cases to 2.0 hours [1.9‐2.4] in the remaining two thirds; *P* < 0.01. In addition, computer scientist and physician spent a total of 35.5 and 33.0 working hours on setting up the digital infrastructure, and on training and testing. Sublobar agreement between all three pipelines was 20%, mean measurement of agreement (kappa) 0.13. Finally, the survey revealed that 53% of epilepsy centers in Germany currently use ESL for presurgical evaluation.

**Significance:**

This study provides information regarding expected effort and costs for integration of ESL into an epilepsy surgery program. Low result agreement across different ESL pipelines calls for further standardization.

**Key Points**

- This study provides practical information for epilepsy centers considering clinical use of Electrical Source Localization (ESL).
- Although an initial substantial investment in personnel working hours is required, cost to benefit is predictable within 4 months.
- We report a relatively low concordance of results between different ESL programs, highlighting the need for more standardization.
- A survey in German centers found that half implemented ESL for presurgical evaluation, with costs listed as a main reason preventing use.

## 1 INTRODUCTION

Electric source localization (ESL) is a computational analysis method integrating scalp electroencephalography (EEG) and magnetic resonance imaging (MRI) that assists in localizing epileptic EEG activity within a 3D model of the brain. ESL is most commonly applied to interictal epileptiform discharges (‘spikes’) but can also be used to localize ictal EEG patterns.^1^ In the presurgical evaluation of patients with drug‐resistant focal epilepsy, ESL has been shown to be an accurate and helpful noninvasive diagnostic tool. In a recent meta‐analysis reviewing over 25 studies that examined ESL performance, the diagnostic accuracy of interictal ESL was 74% as defined by surgical resection and postsurgical seizure outcome.^2^ Additional studies have shown that ESL results co‐localize well with recordings from intracranial electrodes.^3^, ^4^ In centers with extensive experience in ESL utilization, ESL has been found to provide non‐redundant information useful for clinical decision‐making, especially for deciding on additional invasive work‐up, planning of intracranial EEG electrodes,^5^, ^6^ or in the assessment of patients with non‐lesional epilepsy.^7^ Another advantage of ESL is that unlike other non‐invasive presurgical tools, e.g., magnetencephalography (MEG) or positron emission tomography (PET), it does not require expensive or highly specialized equipment. ESL is based on EEG and MRI alone, both of which are compulsory elements of presurgical evaluation. Therefore, the International Federation of Clinical Neurophysiology (IFCN) has concluded that there is good evidence for the accuracy of ESL in the presurgical evaluation of epilepsy.^8^

Yet, despite these advantages, ESL is utilized in less than 50% of European epilepsy centers.^9^ Reasons for this have not yet been systematically explored, but according to expert opinion may include a perceived high cost or need for extensive specialized personnel training, a knowledge gap with regard to ESL efficacy and usefulness, and variability in both ESL software packages and computational models used.^10^, ^11^ Several academic, freely available software solutions exist but are not licensed for clinical application. In addition, they may require computational skills and an in‐depth understanding of brain modeling, and choosing between at least eight existing platforms may be difficult.^12^ On the other hand, commercial software packages or ESL providers offer easy‐to‐use solutions, license for clinical use, and expert troubleshooting, but they come with financial expenses. If computation of ESL is completely outsourced to a commercial provider, generation of results takes place in a ‘black box’ for the clinician. Another driver of costs is devoting both medical and information technology (IT) personnel for the set‐up and processing of ESL. The workload required has not yet been assessed. Furthermore, as studies on efficacy and clinical utility have been done in highly specialized epilepsy centers, the amount of time needed per patient in these prior studies may not reflect the real‐world effort required for initial setup at an ESL‐naïve tertiary epilepsy center.

Thus, this prospective study aimed at assessing the effort in both physician and IT working times required for first‐time integration of ESL into the presurgical workflow of a single tertiary epilepsy center. Additionally, we aimed at comparing the concordance of results between two freely available and one commercial software packages. Finally, we conducted an online survey regarding the current state of ESL implementation in all surgical epilepsy centers in Germany. The significance of this study is that it provides practical, real‐life information that may assist in the decision to integrate ESL in the presurgical routine.

## 2 METHODS

### 2.1 Study design

**FIGURE 1:**
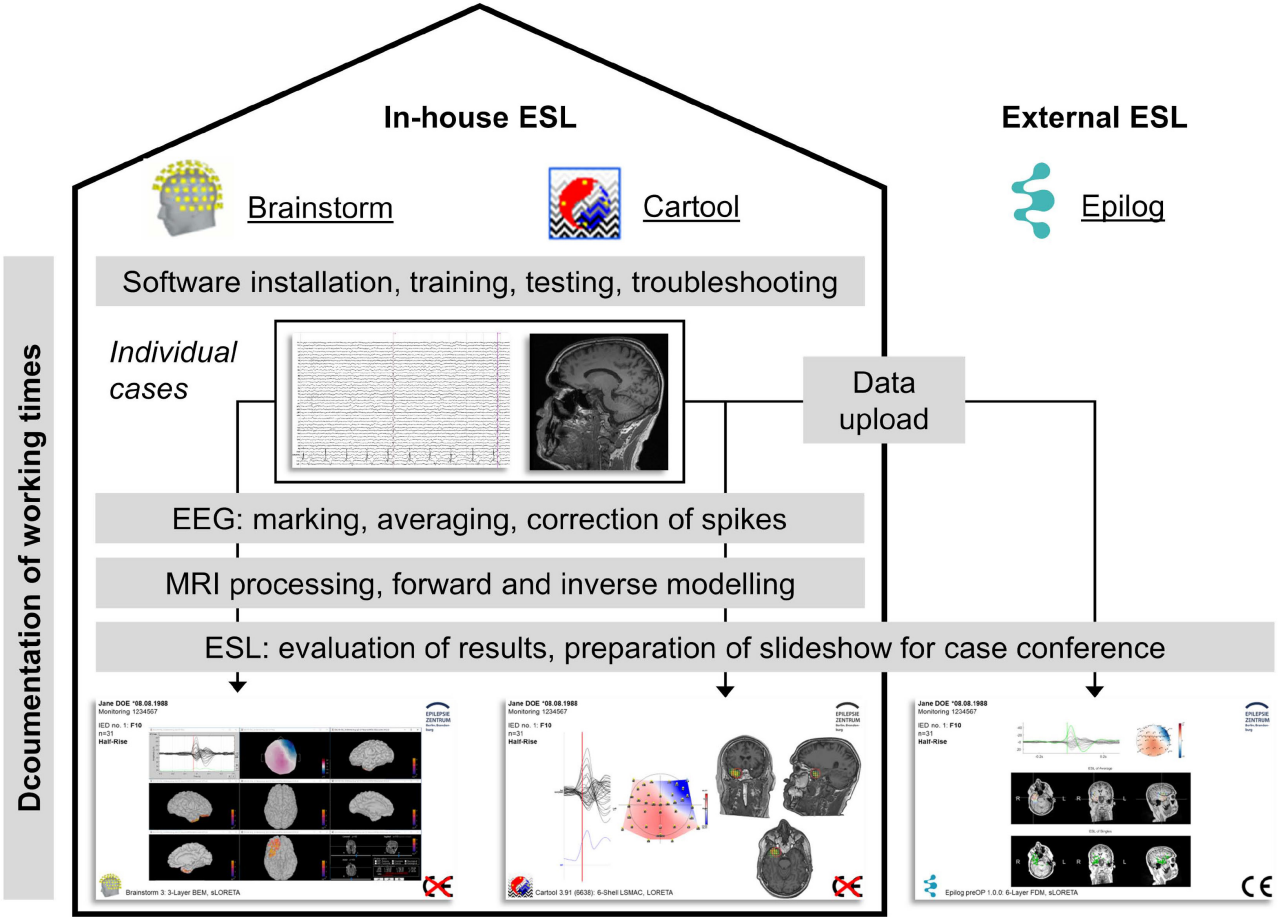
Illustration of the study concept and the EEG source localization (ESL) workflow. ESL was done using three different pipelines, two were in‐house and based on academic freeware (Brainstorm and Cartool), on was external and commercial (Epilog). ESL was based on low‐density EEG and high‐resolution structural MRI. Working times were documented for implementing in‐house ESL and for the processing steps per case (gray).

We conducted a prospective analysis of patients that underwent noninvasive presurgical video EEG monitoring (VEM) between 1st July 2021 and 30th June 2022 at the Epilepsy‐Center Berlin‐Brandenburg (Figure 1). Installation of ESL software and training started on 1st June 2021. The study was approved by the institutional review board at Charité – Universitätsmedizin Berlin (EA2/295/20) and prospectively registered in the German Clinical Trials Register (DRKS00023852).

### 2.2 Patient population

During the study period, patients undergoing presurgical VEM were consecutively recruited. Inclusion criteria were age over 14 years, drug‐resistant focal epilepsy, and noninvasive evaluation for a first epilepsy surgery. Exclusion criteria were presence of a skull defect and lack of written informed consent. For each patient, the following demographic and presurgical evaluation data were collected: sex, age at VEM, epilepsy duration, presence of focal to bilateral tonic–clonic seizures, lateralizing and localizing information based on history and semiology recorded during VEM,^13^ ictal and interictal EEG, findings on MRI and ^18^FDG PET imaging, neuropsychological testing, and conclusions of the multidisciplinary case conference regarding definition of the seizure onset zone and further recommendations.

### 2.3 EEG recording and processing

Video EEG monitoring was carried out at the Institute for Diagnostics of Epilepsy (IDE) as part of the Epilepsy‐Center Berlin‐Brandenburg. The number of EEG surface electrodes was 33‐45, depending on the individual focus hypothesis before VEM. All electrodes recommended by the International Federation of Clinical Neurophysiology were included,^14^ except that T9/10 electrodes were replaced by FT9/10 or sphenoidal electrodes (Sp 1/2; these were not used for ESL). EEG was recorded at sampling rates of 256 Hz, unfiltered, using an in‐house developed EEG software (‘Programm zum Berliner System’, Version 071128; IDE gGmbH). EEG data were stored in 90‐ or 240‐minute files. Using an in‐house script coded by one of the co‐authors (TB), data were converted to the European data format (EDF).

Using average reference and bipolar longitudinal montages, interictal epileptic spikes were visually identified and marked. Homologous spikes, that is, those with the same configuration and the same topographical maximum, were determined visually and grouped to clusters. Per cluster, a minimum of 20‐30 single spikes was required.^15^ If this number was not achievable in about 45 minutes of working time, the number of spikes was considered insufficient for ESL.

### 2.4 MR imaging and processing

All patients underwent 3‐Tesla structural MRI using a Siemens Magnetom Skyra scanner at Charité – Universitätsmedizin Berlin. 3D T1 images were exported in DICOM format and subjected to automated cortical reconstruction and volumetric segmentation using the freely available Freesurfer image analysis suite version 7.1.1.0.1 (http://surfer.nmr.mgh.harvard.edu/)^16^, ^17^ in a Centos 8 Linux Container on a Proxmox Virtual Environment server accessible through X2GO and Windows network file sharing protocol (SMB).

### 2.5 EEG source localization

Interictal ESL was performed using three different approaches, based on two freely available academic software packages (Brainstorm and Cartool) and one commercial pipeline (Epilog). Brainstorm is a collaborative, open‐source application for EEG and MEG analyses which is documented and freely available for download online under the GNU general public license (http://neuroimage.usc.edu/brainstorm).^18^ Cartool is an EEG analysis software programmed by Denis Brunet at the Functional Brain Mapping Lab, University of Geneva, Switzerland.^12^ It is freely available for download online (https://sites.google.com/site/cartoolcommunity). Epilog NV is a commercial provider of CE‐marked and FDA‐cleared EEG analyses seated in Ghent, Belgium (https://www.epilog.care/). All three approaches used individual head models and linear distributed inverse solutions.

Brainstorm version 3.210520 (20‐May‐2021) was running on the MatLab Runtime R2020a (9.8) that is freely available for download online (https://de.mathworks.com/products/compiler/matlab‐runtime.html). Electrodes were co‐registered manually to the individual head surface. Individual boundary element model (BEM) surfaces were generated using the open‐source software OpenMEEG (http://openmeeg.github.io)^19^, ^20^ with 15 000 vertices on the cortical surface. Dipoles were loosely constrained perpendicular to the cortical surface (0.7). The standardized low‐resolution electromagnetic tomography (sLORETA)^21^ served as inverse solution. Using average reference and bandpass filtering at [1:70] Hz plus notch filtering at 50 Hz, 50 seconds of baseline EEG free of evident epileptic spikes served to compute the noise covariance matrix. Five hundred ms‐epochs of homologous spikes were averaged and subjected to ESL. Source maxima at the averaged spike's half‐rise and peak were represented by crosshairs in the MRI viewer of Brainstorm.

In Cartool version 3.91 (6638), electrodes were co‐registered manually to the head surface. A 3D grid of about 6000 solution points was distributed within the gray matter mask. The Locally Spherical Model with Anatomical Constraints (LSMAC)^12^ with age‐adjusted skull thickness served as forward model, and the low‐resolution electromagnetic tomography (LORETA)^22^ was used as inverse model. Five hundred‐ms epochs of homologous spike clusters were averaged and subjected to ESL, using average reference, bandpass filtering at [1:70] Hz, notch filtering at 50 Hz. Tikhonov regularization was used on the level recommended by Cartool. Source maxima at the averaged spike's half‐rise and peak were displayed within the 3D head model.

Continuous EEG files with markers from visual inspection (EDF) and MRI image files (DICOM) were uploaded to the internet portal of Epilog. Individual six‐layer finite‐differences head models (FDM)^23^ and sLORETA^21^ were used as forward and inverse models. Interictal spikes were extracted using the markers provided and, separately, using automated spike detection of Persyst Spike Detector P14. Based on the topographical maximum, homologous spikes were grouped into clusters and averaged. ESL was computed at onset (not regarded for this study), half‐rise, and peak of the averaged spikes. Source maxima were displayed by crosshairs within the MRI in coronal, transversal, and sagittal view. Separate PDF reports were generated for spikes based on visual marking and those identified by automated spike detection. In cases with interictal ESL, up to 12 selected seizures were additionally subjected to ictal ESL by Epilog, see details in Appendix S1.

Interictal ESL with Brainstorm and Cartool was performed by BJV on a customary PC with 3.20 GHz processor, 8.00 GB installed RAM, Windows 10 Professional x64 bit system, and a single 24‐inch monitor. Since Brainstorm and Cartool are not licensed for clinical use, only Epilog results were regarded for clinical decision‐making.

### 2.6 Assessment of ESL concordance

Concordance of different interictal ESL pipelines were assessed by subjecting identical spikes to all three programs. To reduce bias, results were evaluated independently by different authors, with Brainstorm and Cartool results assessed by BJV and interictal Epilog results evaluated by GM. Ictal ESL was evaluated by KG. ESL results were visually assessed on sublobar (19 per hemisphere), lobar, and hemispheric levels.^24^ Concordance to visual EEG analysis was assessed on lobar and hemispheric levels.

### 2.7 Recording of working times

Both authors BJV (neurologist) and TB (computer scientist) documented their working times for implementing ESL and analyzing all eligible cases during the 12‐month study period. Working times were recorded separately for the different software packages and for the different working steps. Tasks independent from patient cases included (1) installation of software, (2) training and testing, (3) troubleshooting, and (4) others. Working steps related to patient cases included (a) processing of the MRI, (b) forward and inverse modeling, (c) marking of spikes, (d) averaging and correction of spikes, (e) evaluating ESL results and preparing a slideshow with screenshots, and (f) others like loading, editing, and zipping files.

Working times for each step were documented in minutes and could be rounded to 5‐minute blocks. To obtain results independent from computational power, only time effectively spent working on ESL was documented. Waiting times during computation that could be spent on other tasks were not counted as working times.

Before June 2021, TB had not been working with ESL or with any of the software packages used. BJV had used Cartool^24^, ^25^ and Epilog^26^ for retrospective, scientific ESL analyses in another epilepsy center. He had not used ESL for prospective evaluation of patients, nor had he used Brainstorm. To become familiar with Brainstorm, he used the official online tutorials (https://neuroimage.usc.edu/brainstorm/Tutorials).

### 2.8 Online survey

As an addendum to the main study, an online survey was conducted to evaluate the current state of ESL in presurgical epilepsy evaluation and common barriers in Germany in April 2022. Nineteen epilepsy centers with epilepsy surgery programs were identified through the website of the German Society of Epileptology (DGfE) and personal knowledge. An invitation to participate in the survey was sent via e‐mail to the head of each institution and to the head of the respective presurgical evaluation program. It was asked that one person per center completed the survey. Naming the institution was optional; otherwise, the survey was anonymous. If provided, centers' names were separated from all remaining answers before analysis. The survey was cleared by the data protection support team at Charité – Universitätsmedizin Berlin; survey structure and results are detailed in Table S2.

### 2.9 Data capture and statistical analysis

Pseudonymized patient data, ESL results, and working times were recorded and managed using Research Electronic Data Capture (REDCap) tools hosted at Charité – Universitätsmedizin Berlin.^27^, ^28^ Likewise, the online survey was conducted using a REDCap tool.

The measure of agreement between results of different ESL pipelines was calculated by using unweighted Cohen's kappa (*κ*) for agreement between two software programs, and unweighted Fleiss' *κ* for determining agreement between three software programs. Values between 0‐0.2 reflect slight agreement, 0.21‐0.4 fair agreement, 0.41‐0.6 moderate agreement, 0.61‐0.8 substantial agreement, and 0.81‐1 almost perfect agreement.^29^, ^30^

Continuous variables are presented as median [interquartile range] and tested with Mann‐Whitney *U* test due to skewed data distributions. Categorical variables were tested with Chi‐square test. Statistical analysis was performed with SPSS Statistics version 28.0 (IBM). To account for testing multiple variables, statistical significance was set at *P* < 0.01.

### 2.10 Ethical publication statement

We confirm that we have read the Journal's position on issues involved in ethical publication and affirm that this report is consistent with those guidelines.

## 3 RESULTS

### 3.1 Patient population and presurgical work‐up

Forty consecutive patients were eligible for the study, and all agreed to participate. Nineteen were females; age at evaluation ranged from 15 to 61 years. For details, see Table 1, Tables S1 and S2, and Figures S1 and S2. Twenty‐two patients (55%) could be analyzed with interictal ESL while the remaining 18 patients had either too few interictal epileptic spikes or polyspikes which could not be averaged. Besides interictal epileptic activity, there were no significant demographic or clinical differences between patients with and without interictal ESL. Overall, presurgical work‐up resulted in conclusive hypotheses regarding focus lateralization in 31 patients (78%) and localization in 28 (70%). Surgery was recommended in 13 patients (33%), while 15 (38%) and 12 (30%) were recommended additional noninvasive or intracranial diagnostics.

**TABLE 1:** Patient characteristics.

	All patients	ESL analyzed	ESL not possible
n	40	22	18
Sex, male	21 (53%)	12 (55%)	9 (50%)
Age, years	29.5 [21.0–43.5]	29.0 [19.0–44.5]	30.5 [26.3–42.5]
Epilepsy duration, years	14.0 [6.8–26.0]	13.0 [6.3–26.0]	16.5 [7.8–25.8]
Presence of bilateral tonic clonic seizures	32 (80%)	18 (82%)	14 (78%)
VEM results			
Number of seizures	6 [2–13]	9 [3–14]	3 [1–9]
Patients with multiple seizure foci	15 (38%)	10 (45.5%)	5 (28%)
Patients without seizures	5 (13%)	1 (5%)	4 (22%)
Patients without epileptic interictal discharges	11 (28%)	0	11 (61%)
Case conference recommendations			
Final decision‐ surgery/no surgical candidate	7 (18%)/6 (15%)	4 (18%)/4 (18%)	3 (17%)/2 (11%)
Further evaluation required‐ invasive/non‐invasive	12 (30%)/15 (38%)	9 (41%)/5 (23%)	3 (17%)/10 (56%)

### 3.2 Time invested in ESL

**FIGURE 2:**
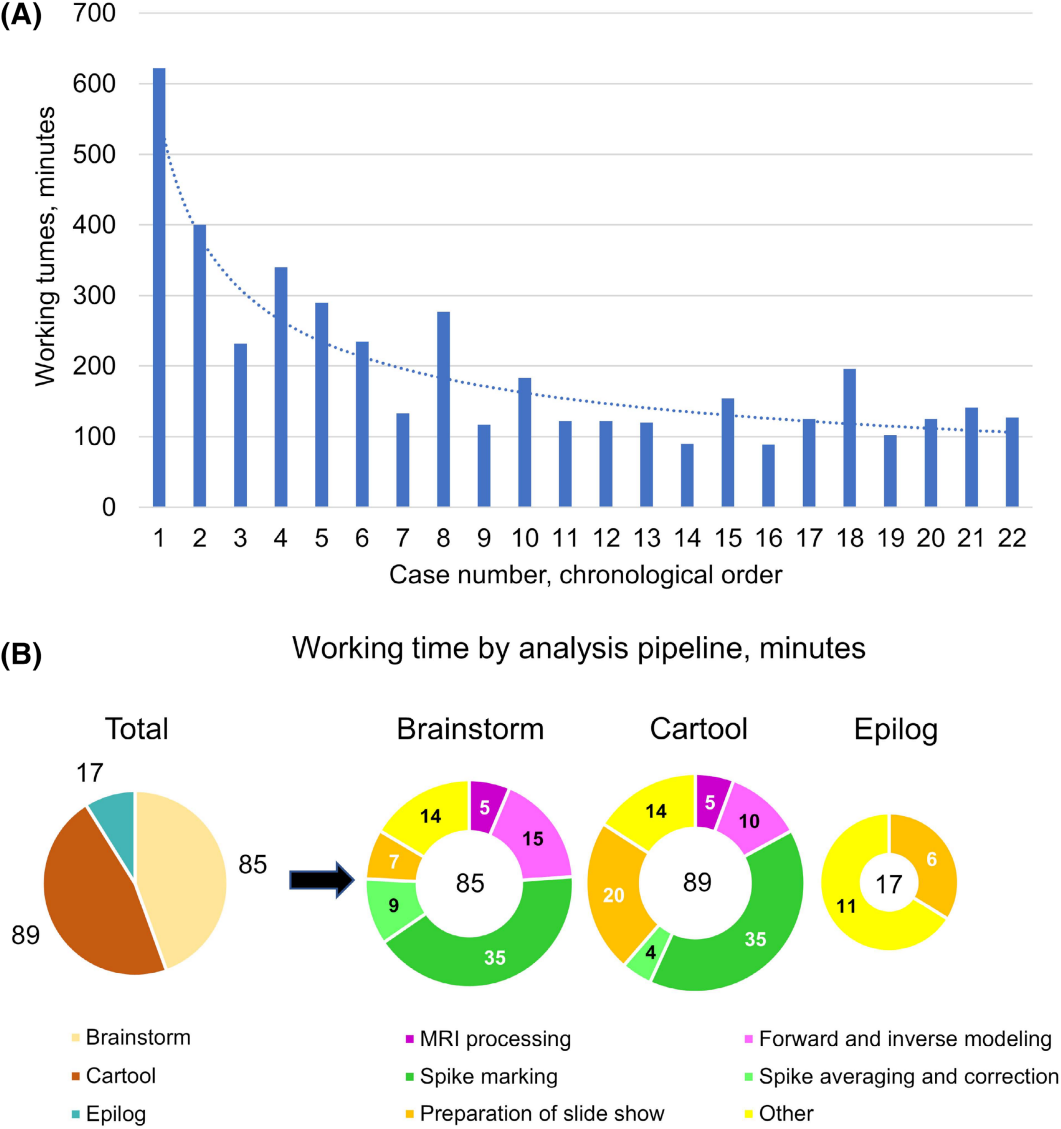
(A) Time spent on electric source localization (ESL) per case in chronological order. (B) Total average working times for the separate processing steps, required for analysis of the last eight cases of the study, by pipeline (Brainstorm, Cartool, and Epilog).

During the study period (n = 40), an overall total of 147.1 working hours were spent on ESL by the physician (111.6 hours) and the computer scientist (35.5 hours). For successfully analyzed cases (n = 22), a total of 72.4 hours (median per case of 2.3 hours [2.0‐4.1]) was required by the physician. As the study progressed, processing times became significantly shorter, with the learning curve stabilizing after case #8. Accordingly, the first third of patients required a significantly longer processing time of 4.7 hours median [3.9‐6.4] compared to the remaining patients requiring 2.0 hours [1.9‐2.4]; *P* = 0.002 (Figure 2A). Furthermore, 6.2 additional hours (median 0.1 hours [0‐0.6] per patient) were spent on cases ultimately not eligible for ESL (n = 18), mainly for reviewing their EEG.

In addition to time directly invested in patient analysis, 33 hours (median 0.9 hours [0.1‐3.3] per month) were spent by the physician in training, testing, and troubleshooting ESL software, and 35.5 hours (median 0.5 hours [0‐3.8] per month) were spent by the computer scientist in setting up system infrastructure and solving technical problems. Again, general ESL training and set‐up were largely done in the initial third of the study period for both the physician (3.7 hours [1.4‐14.6] per month, compared to 0.3 hours [0‐1.0], *P* = 0.002) and the computer scientist (5.6 hours [3.4‐13.9] vs 0.2 h [0‐0.8], *P* = 0.002).

In comparing working times devoted to each ESL software, the use of Brainstorm and Cartool required 38 minutes [27‐103] and 40 minutes [30‐66] per case; *P* = 0.6, in addition to a median of 40 minutes [29‐53] that were required for review and marking of the EEG. Using Epilog's service required significantly less time of 10 minutes [10‐30], *P* = 0.003. Other than spike marking, the most time‐consuming processing step for the freeware pipelines was forward and inverse modeling requiring a median of 29 minutes [25‐55]. With Epilog, preparation of a slideshow for presentation was the most time‐consuming task requiring a median of 5 minutes [5‐10]. The time spent on each processing step per case, as was recorded in the final third of the study, is presented in Figure 2B.

### 3.3 Concordance of results between different ESL pipelines

**FIGURE 3:**
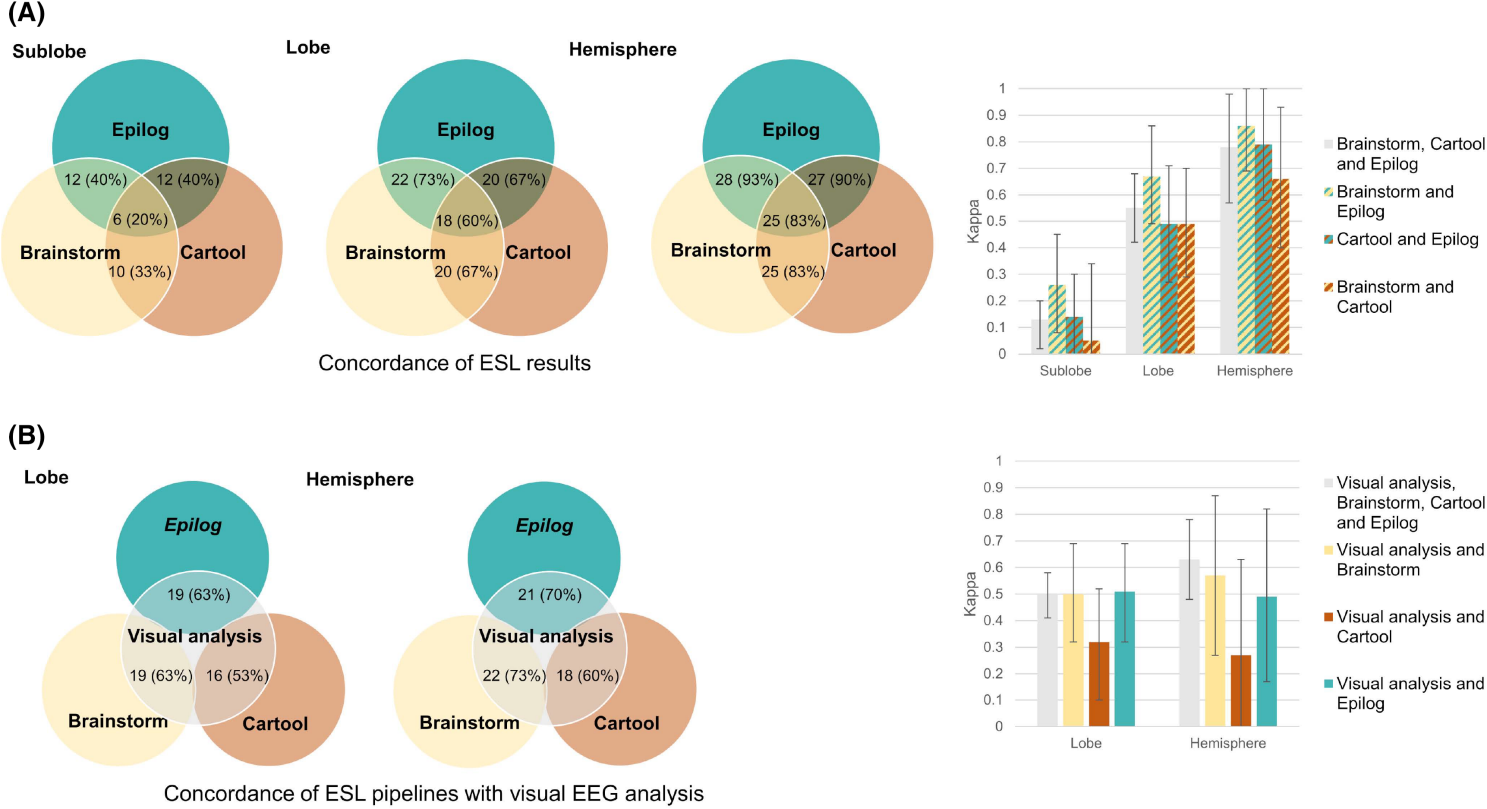
(A) Concordance of ESL results obtained with the different pipelines. Venn diagrams show overlap of results between ESL pipelines on the sublobe, lobe, and hemispheric level. Column charts show kappa agreement levels between ESL pipelines. (B) Concordance of ESL pipelines with visual analysis of the EEG is shown on the lobe and hemispheric level. Kappa agreement levels are shown between ESL pipelines and visual analysis of EEG.

In the 22 patients eligible for interictal ESL, a total of 30 spike clusters were examined. The median number of single spikes per average was 28 [22‐33]. At the averaged spike's half‐rise, sublobar concordance of all three ESL software pipelines was achieved in six spikes (mean measurement of agreement *κ* = 0.13), whereas agreement on lobar and hemispheric levels were achieved in 18 (*κ* = 0.55) and 25 spikes (*κ* = 0.78), respectively. In examining agreement between different freeware and the commercial ESL pipeline, there were no statistically significant differences on the sublobar level (Brainstorm and Epilog, 12 spikes (*κ* = 0.26), Cartool and Epilog 12 spikes (*κ* = 0.14), Cartool and Brainstorm 10 spikes (*κ* = 0.05)). For additional details, see Figure 3A.

Comparing interictal ESL and visual spike analysis, results obtained with Brainstorm and Cartool agreed with visual analysis in 19 (*κ* = 0.50) and 16 (*κ* = 0.32) of 30 spikes on the lobar level, and in 22 (*κ* = 0.57) and 18 spikes (*κ* = 0.27) on the hemispheric level, respectively. Epilog results were concordant with visual analysis on the lobar level in 19 spikes (*κ* = 0.51) and on the hemispheric level in 21 spikes (*κ* = 0.49) (Figure 3B).

In addition to manually marked spikes, in seven of the 22 patients (32%), Epilog's automated spike detection resulted in identification of an additional spike type with a maximum >1 electrode away from that of a manually marked spike, possibly adding additional, previously undetected EEG information. Ictal ESL results, as well as concordance of results for interictal ESL performed at the peak of the averaged spike, are reported in the Appendix S1.

### 3.4 ESL implementation in tertiary epilepsy centers in Germany

All 19 contacted surgical epilepsy centers in Germany completed the survey (100% participation rate; Table S2). Ten centers (53%) reported use of ESL in presurgical clinical routine: Six centers (32%) use ESL regularly whereas the remaining four (21%) apply it on selected cases only; two centers report use in non‐lesional or complex cases, and two other centers (including ours) stated that results are obtained for research purposes but not yet for clinical decision making. Five centers (50%) use commercial software only, four centers (40%) use both commercial and freely available, academic software, and one center exclusively uses freely available software. Among the nine centers not using ESL, five (56%) had previous experience using ESL and have discontinued use. Reasons for not applying ESL were cost or staff shortage in five centers (56%), whereas the remaining four (44%) perceived a lack of additional benefit. Notably, these four centers had prior experience with ESL. For statements regarding the use of MEG source localization (see Appendix S1).

## 4 DISCUSSION

In epilepsy centers with a long experience in ESL, this technology has been shown to provide non‐redundant clinical information, directly influencing decision making in a third of patients evaluated for epilepsy surgery.^5^, ^6^ On the other hand, despite multiple commercial and freely available pipelines, ESL is still underutilized by the majority of surgical epilepsy centers. In this prospective study, we addressed a known common barrier to integration of new medical technology: the expenditure of time.^31^ We found that on the one hand, a significant initial investment in time is required from both the medical and IT side. On the other hand, after a short introductory period, computing ESL requires moderate additional investment. Furthermore, we report that only half of patient cases appear eligible for ESL, and we show a relatively low concordance of results among the different pipelines used. Finally, a survey among epilepsy centers in Germany revealed that ESL is used in half of them only, confirming earlier surveys in Europe.^9^

Of the 40 patients that were recruited to the study, 45% were not eligible for interictal ESL because of too few spikes or polyspikes. This is on the higher end of the spectrum seen in prior prospective studies, in which the range of excluded patients due to lack of sufficient interictal activity was 0%‐58%.^6^, ^32^, ^33^, ^34^, ^35^ This may be due to our study population with at least 18% of cases with suspected extratemporal epilepsy and another 30% with unclear localization after noninvasive diagnostics, since extratemporal epilepsy is known to yield less interictal EEG activity than temporal lobe epilepsy.^36^ Furthermore, we used a standard 33‐45 electrode set‐up rather than high‐density EEG with face coverage as used in some prior ESL studies.^26^ We also canceled the EEG review if a sufficient number of spikes could not be found within approximately 45 minutes. Notably, spikes were marked by an experienced epileptologist, and EEGs were previously reviewed by other senior epileptologists. This reflects real‐life conditions which are important for cost–benefit analysis of centers considering integration of ESL.

In examining the time invested by both the physician and computer scientist during the study period, a clear learning curve was seen, stabilizing after approximately eight patients, correlating in this study to 4 months. This suggests that even if an initial substantial investment is needed in terms of both medical and IT working hours, the cost–benefit balance is predictable after a short period of time. With spike marking being the most time‐consuming single step, working times directly depend on the minimum number of spikes considered necessary for ESL. Notably, usage of the commercial ESL service required significantly less working time than using the freeware pipelines which is certainly due to the fact that all computation was performed externally rather than in‐house. This may be an advantage in centers lacking personnel but may pose a disadvantage in terms of less transparency regarding the underlying processing steps as compared to in‐house computation. It should be noted that the workflow for customers of Epilog NV has changed since the study was conducted.

Our study was neither aimed at nor powered for assessing the diagnostic accuracy of various ESL tools, and clinical outcome measures like postsurgical seizure outcome are not yet available for our cohort. Nevertheless, we compared the results obtained with the different pipelines to each other and found slight agreement only (*κ* = 0.13 on the sublobar level). Although all ESL approaches have been previously validated for clinical and research purposes,^32^, ^37^, ^38^, ^39^, ^40^, ^41^, ^42^ the use of different outcome measures and electrode arrays (high vs low density) in prior studies limits the direct comparison of different ESL pipeline performance during initial integration at an epilepsy center. One prospective study comparing two commercial in‐house ESL solutions reported moderate agreement (*κ* = 0.43 using distributed source models).^6^ Moreover, it has been systematically established that the choice of a specific inverse solution algorithm and software package significantly impacts the results of ESL.^43^ Since different ESL pipelines lead to differing results, application of several methods in parallel seems wise.^44^, ^45^ Furthermore, to reduce variability in software packages and models used, there is an urgent need for standardized guidelines for the use of ESL in presurgical evaluation.

Finally, to give additional updated context to current barriers of ESL integration, we conducted a survey among surgical epilepsy centers in Germany and report that only half of them use ESL for presurgical epilepsy evaluation. For comparison, nine of 24 evaluated European centers (38%) used ESL in 2014.^9^ Interestingly, half of the centers that currently do not use ESL have been using it in the past, but the method has not been able to establish itself sustainably. Costs were listed as a major reason for non‐usage, suggesting that affordable solutions are necessary. This is despite the fact that ESL does not require additional expensive equipment, such as PET or MEG.

This study had several limitations. First, in‐house ESL was performed by a single physician (BJV) who had prior training in ESL for scientific purposes, while the commercial analyses were conducted externally in a blinded manner. A potential bias toward higher concordance between the two in‐house pipelines (Brainstorm and Cartool) compared to the external pipeline (Epilog) cannot be ruled out, although pairwise agreement rates were lowest between Brainstorm and Cartool. Second, because BJV had already been familiar with Cartool and Epilog, physician working hours are certainly underestimated. Nevertheless, a learning curve became evident, and working hours at the end of the study period can be considered reliable. Finally, only one head model and one distributed inverse solution were used per pipeline, whereas single dipole models were not regarded at all. This approach limits the generalizability of ESL results but reflects real‐life conditions, and systematic comparison of more variables would not have been realistic.

In conclusion, this study shows that implementing ESL in presurgical epilepsy evaluation is feasible in formerly ESL‐naïve centers. One physician with basic understanding of ESL theory and practice, and one computer scientist seem sufficient. This study reports cost in personnel working hours required for initial clinical integration of ESL, providing helpful information for centers interested in use of this technology. Low concordance of results obtained with different software programs and heterogenous importance of the technology in surgical epilepsy centers in Germany highlight the need for additional evidence and more standardization.

## CONFLICT OF INTEREST STATEMENT

MH received speaker's honoraria and/or consultancy fees from Angelini, Bial, Desitin, Eisai, Jazz, Neuraxpharm, UCB, and Zogenix within the last 3 years. All other authors declare that they have no competing interests.

## Supporting information


Table S1

Table S2

Appendix S1
