# Supplementary material for: Integration of interictal EEG source localization in presurgical epilepsy evaluation – A single‐center prospective study
Source: Epilepsia Open. 2023 May 19;8(3):877–87. doi: 10.1002/epi4.12754 (PMC10472400; doi:10.1002/epi4.12754)
Supplement: Supplementary file 3 — Appendix S1 [file EPI4-8-877-s002.pdf]

Appendix S1

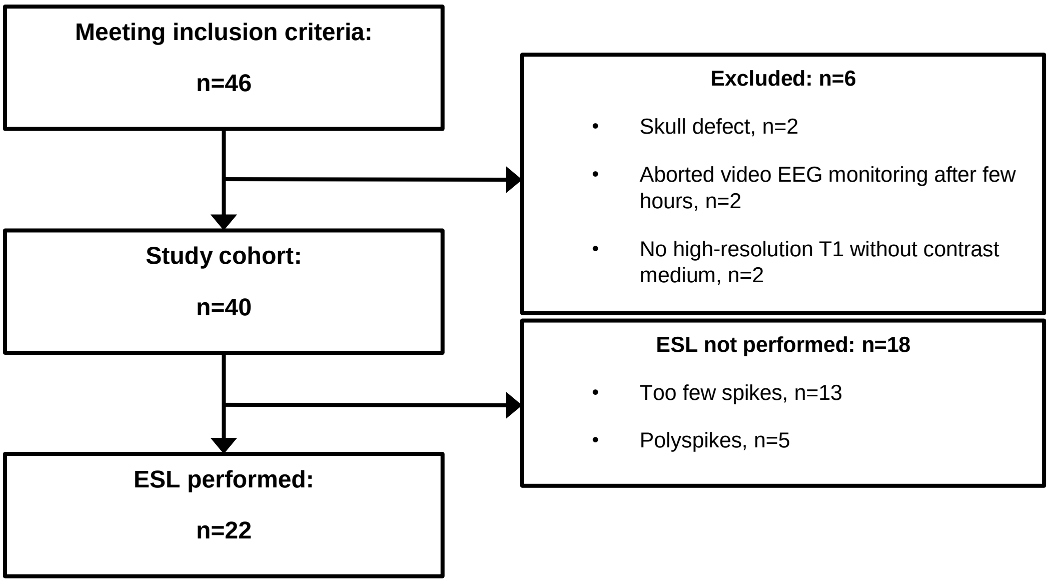

Supplementary figure 1. Flow chart of patients included in study.

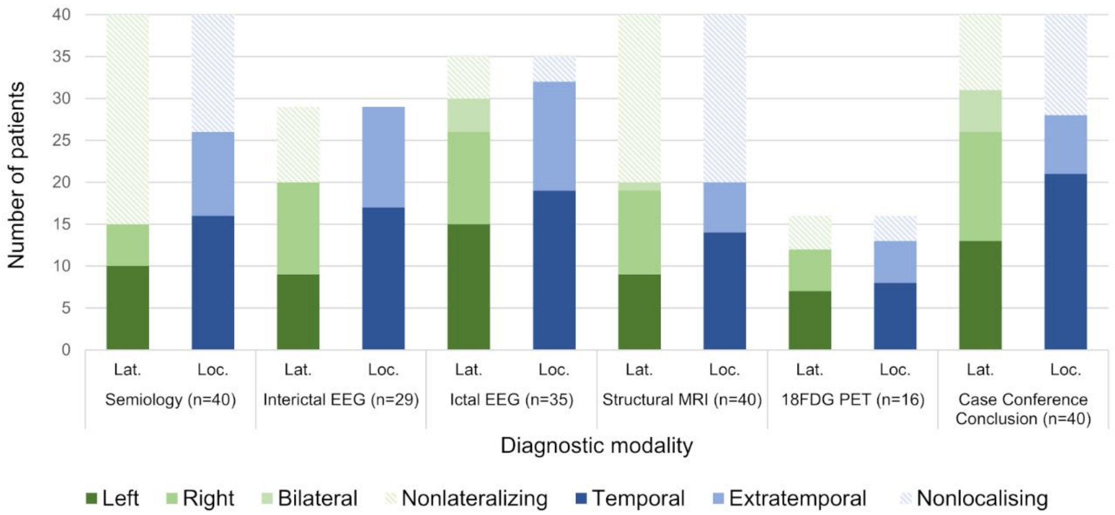

Supplementary figure 2. Results of presurgical noninvasive diagnostic workup. For each diagnostic modality, lateralization (Lat.) and localization (Loc.) results are presented. There were no statistically significant differences between patients that underwent ESL analysis and those that did not.

Sub-analysis of concordance between ESL pipelines based on clinical factors

We conducted an additional sub-analysis to examine the concordance of ESL based on suspected epilepsy localization and the presence of lesional imaging. We observed higher agreement in temporal lobe (n=18 spikes) cases compared to extratemporal cases (n=12 spikes; sublobe: $\kappa=0.12$ vs. $\kappa=0.08$, $p=0.75$; lobe: $\kappa=0.7$ vs. $\kappa=0.37$, $p=0.02$; hemisphere: $\kappa=0.85$ vs. $\kappa=0.63$, $p=0.35$). In cases with lesional (n=12 spikes) versus non-lesional (n=18 spikes) imaging, we found overall higher agreement among lesional patients (sublobe: $\kappa=0.35$ vs. $\kappa=0.04$, $p=0.004$; lobe: $\kappa=0.71$ vs. $\kappa=0.45$, $p=0.08$; hemisphere: $\kappa=0.88$ vs. $\kappa=0.7$, $p=0.4$).

Concordance of results between different ESL pipelines performed at peak of the spike

In the 22 patients eligible for interictal ESL, a total of 30 spike clusters were examined. At the averaged spike's peak, sublobar concordance of all three ESL software pipelines was achieved in 10 spikes (mean measurement of agreement κ=0.27), whereas agreement on lobar and hemispheric levels were achieved in 18 (κ=0.55) and 25 spikes (κ=0.78), respectively. In examining agreement between different freeware and the commercial ESL pipeline, there were no statistically significant differences on the sublobar level (Brainstorm and Epilog, 12 spikes (κ=0.26), Cartool and Epilog 11 patients (κ=0.14)). For additional details, see supplementary figures 3 and 4.

Comparing interictal ESL and visual spike analysis, results obtained with Brainstorm and Cartool agreed with visual analysis in 19 (κ=0.50) and 12 (κ=0.32) of 30 spikes on the lobar level, and in 22 (κ=0.57) and 18 spikes (κ=0.27) on the hemispheric level, respectively. Epilog results were concordant with visual analysis on the lobar level in 19 spikes (κ=0.55) and on the hemispheric level in 21 spikes (κ=0.49); supplementary figure 4.

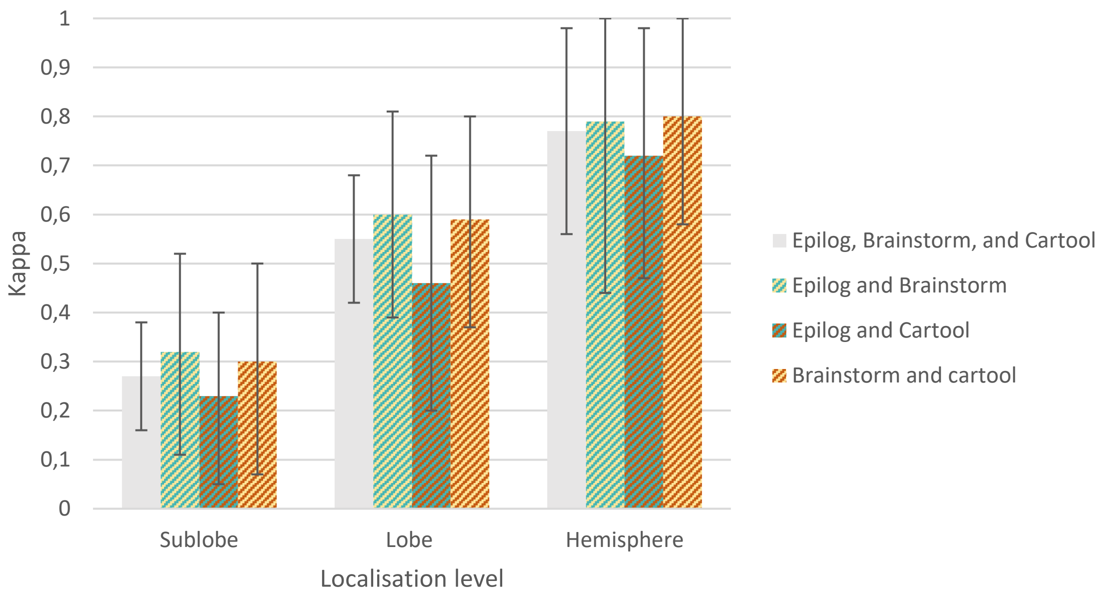

Supplementary figure 3. Concordance of ESL results at the spike peak on sublobar, lobar, and hemispheric levels between different electric source localization pipelines.

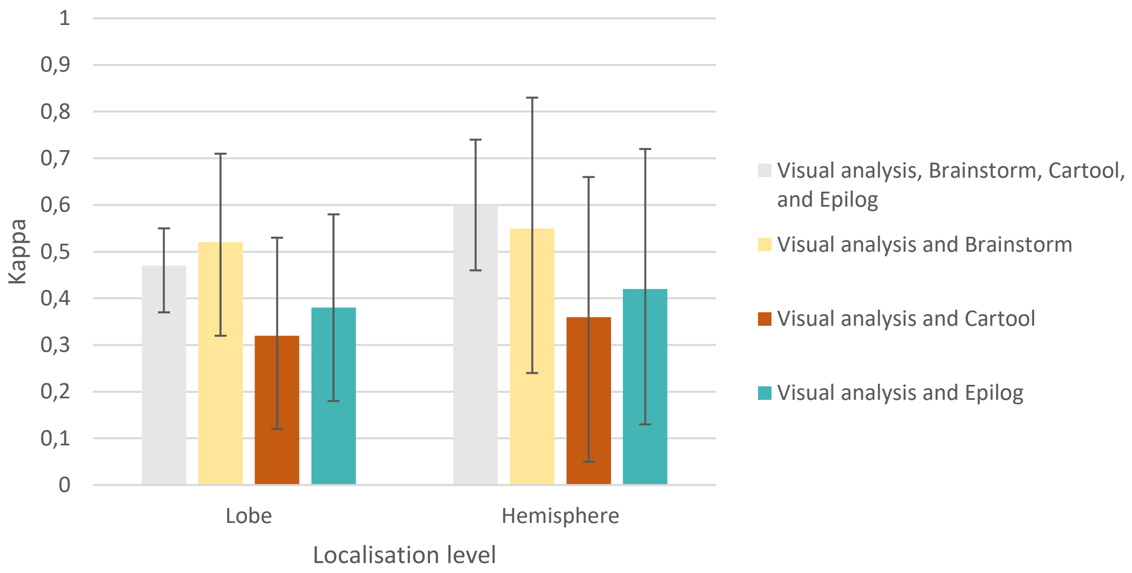

Supplementary figure 4. Concordance of ESL results at the spike peak on lobar and hemispheric level between different electric source localization pipelines and visual analysis of EEG.

Comparison of results between manually submitted and automatically identified spikes

When comparing ESL results from manually submitted and automatically identified spikes that were maximal at the same electrode, at the spikes half peak localization results were concordant on the sublobar level for 16 spikes (κ=0.6) and on the lobar and hemispheric levels for 21 spikes (κ=0.9), demonstrating overall high concordance. In addition to the manually submitted spikes, Epilog detected 7 extra spikes not provided by us.

Ictal ESL analysis

Eighteen patients that underwent interictal ESL analysis were also subjected to ictal ESL analysis done by Epilog. Per patient, up to 12 seizures were selected for ictal ESL, resulting in a total of 79 analyzed seizures. Seizure onset was marked manually by BJV. Within a window of interest ranging from 2 s before until 5 s after the marker, six 2-second epochs were analyzed. Per epoch, the two most dominant frequency bands were isolated and subjected to ESL. Fifty-five seizures (69.6%) of 16 patients (88.9%) yielded meaningful ESL ictal results. Among these, ESL and visual analysis were concordant with respect to both side and lobe in 33 seizures (60%).

Results of survey - Utilization of Magnetic Source Localization (MSL)

From all 19 contacted surgical epilepsy centers in Germany, one person completed the survey (100% participation rate). Eleven centers treat both adult and pediatric population, six treat adults only, and two treat pediatric patients only. Eight centers (42%) reported use of MSL in presurgical clinical routine. Two centers (11%) use MSL regularly, whereas in the remaining six centers (32%) it is applied only in selected cases. Four centers use MSL in non-lesional cases, two centers in cases of inconclusive noninvasive evaluation, and one center for research purposes. Four centers process MEG internally using commercial software, and five centers do so with freeware. Six vs. two centers rate MSL as requiring high vs. low effort, likewise in six vs. two centers it is considered of low vs. high benefit. Amongst 11 centers not utilizing MSL, 6 centers have used it previously, and in all centers the reason for not using this tool is that MEG is not available.
